# Supplementary material for: Glutamate receptor composition at Drosophila neuromuscular junctions depends on developmental stage and muscle identity
Source: eLife. 2026 Oct 30;15:RP111371. doi: 10.7554/eLife.111371 (PMC13722416; doi:10.7554/eLife.111371)
Supplement: Peer review history

# Editor report: eLife Assessment

**DOI:** 10.7554/eLife.111371.3.sa0

**Authors:** Maarten F Zwart

**Affiliations:** University of St Andrews United Kingdom

**Keywords:** Convincing, Important

This study provides **important** findings on the composition of glutamate receptor (GluR) subunits across diverse *Drosophila* neuromuscular junctions, spanning larval and adult stages and multiple muscle types. Most previous work has focused on larval body wall muscles, and this study demonstrates that this model does not capture the full diversity of GluR composition across development and tissues. The revised manuscript provides a **convincing** account of this diversity and will be of broad interest to researchers across neuroscience.

# Referee report: Reviewer #1 (Public review)

**DOI:** 10.7554/eLife.111371.3.sa1

Summary:

The manuscript by Sustar et al. takes a methodical approach to document the types of glutamate receptor subunits that reside in *Drosophila* muscles, examining developmental stages spanning from larvae to adults. Prior work thoroughly documented the subunits operating in *Drosophila* larval body wall muscles. Most subsequent research focused on the glutamate receptor heterotetramers found in the body wall, composed of GluRIIA/C/D/E or GluRIIB/C/D/E subunits, along with auxiliary subunits like isoforms of Neto.

For the current work, the authors report that the larval muscle glutamate receptor composition is not universal for all *Drosophila* muscles. They examine the following muscle systems: larval body wall, adult abdomen, adult leg coxa, and adult indirect flight. They also briefly examine adult muscle structures associated with the proboscis, neck, and haltere. The authors find that the receptor subunits in the adult abdomen (mostly) match those in the larval body wall. This makes sense given that the adult abdominal muscles are derived from the larval body wall. Yet not much else matches the larval body wall. For example, all (or most) of the GluRII-type subunits are missing from the adult indirect flight muscles. Leg muscles have GluRII-type subunits, but they do not have all of them expressed prominently, and they are missing GluRIIB. Additionally, leg muscles express a glutamate-gated chloride channel, which could be a source of inhibitory glutamatergic transmission. Interestingly, when it comes to non-abdominal adult muscles, one general theme seems to be an active promoter (GAL4 driver) for the kainate-type glutamate receptor called Clumsy. The authors propose that Clumsy could be key to understanding how functional GluR complexes are assembled in adult insects.

Strengths:

(1) Documenting the types of glutamate receptors that operate in diverse insect muscle systems is important because it uncovers fundamental information.

(2) Much of the prior research focus has been on how the body wall muscle tetramers assemble and operate. It is a strength to demonstrate the other receptor solutions used by adult NMJs.

(3) The work uses GAL4 drivers and immunohistochemistry (when possible) in combination to draw conclusions.

(4) The muscle anatomical analyses are high quality. This allows the research group to reach refined conclusions.

(5) The confocal-level images of synaptic active zones and their apposed glutamate receptor clusters are high quality.

(6) The dataset adds specific detail and complementary context to bulk transcript analysis collections like FlyAtlas.

(7) The Discussion section is insightful. It is not simply a recap of the data in the paper. It is also an integration of that data with prior work, and it suggests direct experiments that the research team or others could attempt downstream.

Weaknesses:

One can draw expression-level conclusions from these data. But genetic tests (e.g., would clumsy losses of function impair leg muscles?) could help the authors and the field draw stronger conclusions about the roles of some of these glutamate receptor gene products.

Overall Assessment and Discussion:

The data in this study are high quality, and the results support the main conclusion: adult muscle glutamate receptor clusters do not recapitulate the "canonical" larval body wall clusters.

Comments on revised version.

The authors have done a serious revision of their manuscript, and they have directly addressed context/interpretation-level concerns that I had from the first round.

# Referee report: Reviewer #3 (Public review)

**DOI:** 10.7554/eLife.111371.3.sa2

The Sustar et al. manuscript catalogs glutamate receptor composition across distinct *Drosophila* NMJs: larval and adult abdominal NMJs, as well as NMJs on adult leg and flight muscles. This work is important and probably overdue. The larval NMJ is the exemplar NMJ in this system, and the identity of "essential" and "alternative" subunits at this stage is assumed by many to hold across developmental stages and NMJ types. Here, the authors show that there is surprising diversification among NMJ types and that the notion of essential/alternative subunits only holds true at larval NMJs.

The study will generate interest in the Clumsy GluR subunit, which has not been well-characterized at all, but is widely expressed at adult NMJs. They also find striking extrasynaptic expression of glutamate-gated chloride channel GluRClalpha in adult leg and flight muscles, raising questions about its role. The study is interesting, logical, and well-written. The figures are clear, and the discussion was particularly thoughtful.

Comments on revised version.

This is a nicely revised manuscript that went a long way toward addressing the concerns in the first round. The response to the reviewers is clear and thoughtful. I think the question is framed very well and the authors do a much better job throughout making clear where the heterologous approaches agree and differ. I also think the Limitations section in the Discussion does a great job putting the work in context.

# Author comment: Author response

**DOI:** 10.7554/eLife.111371.3.sa3

**Authors:** Anne Sustar, Chengjie Qiu, Yu Xiong, Dion K Dickman, John C Tuthill

**Affiliations:** University of Washington Seattle United States; University of Southern California Los Angeles United States; University of Southern California Los Angeles United States; University of Southern California Los Angeles United States; University of Washington Seattle United States

The following is the authors’ response to the original reviews.

**eLife Assessment:**

This study provides important findings on the expression of glutamate receptor (GluR) subunits across developmental stages and muscle types in *Drosophila*. It shows that adult muscle differs in GluR composition from larval body wall muscles, which have been the focus of most past studies. The study, while convincing, could be strengthened by acknowledging that it relies on heterogeneous methods and the absence of positive signals to infer receptor loss, which limits confidence in some of its claims. The findings illuminate how *Drosophila* excites muscles in diverse tissue types at different life stages, and are of interest to researchers across neuroscience.

A note on the eLife assessment

We are grateful for the assessment and for the reviewers' engagement. The assessment notes that the study "could be strengthened by acknowledging that it relies on heterogeneous methods and the absence of positive signals to infer receptor loss." We have taken this seriously and have addressed it in three ways in the revised manuscript:

(i) We have replaced claims of absence with "not detected" throughout;

(ii) We have added a paragraph describing the discrepancies between methods and the specific false-negative risks of each; and

(ii) We have made explicit the internal positive controls that constrain our negative results, including the reciprocal GluRIIB/GluRIIC labeling within a single femur and the detection of Brp, Neto-β, and GluClα in the same flight muscle preparations in which GluRII subunits were not detected.

**Public Reviews:**

**Reviewer #1 (Public review):**

Summary:

The manuscript by Sustar et al. takes a methodical approach to document the types of glutamate receptor subunits that reside in *Drosophila* muscles, examining developmental stages spanning from larvae to adults. Prior work thoroughly documented the subunits operating in *Drosophila* larval body wall muscles. Most subsequent research focused on the glutamate receptor heterotetramers found in the body wall, composed of GluRIIA/C/D/E or GluRIIB/C/D/E subunits, along with auxiliary subunits like isoforms of Neto.

For the current work, the authors report that the larval muscle glutamate receptor composition is not universal for all *Drosophila* muscles. They examine the following muscle systems: larval body wall, adult abdomen, adult leg coxa, and adult indirect flight. They also briefly examine adult muscle structures associated with the proboscis, neck, and haltere. The authors find that the receptor subunits in the adult abdomen (mostly) match those in the larval body wall. This makes sense given that the adult abdominal muscles are derived from the larval body wall. Yet not much else matches the larval body wall. For example, all (or most) of the GluRII-type subunits are missing from the adult indirect flight muscles. Leg muscles have GluRII-type subunits, but they do not have all of them expressed prominently, and they are missing GluRIIB. Additionally, leg muscles express a glutamate-gated chloride channel, which could be a source of inhibitory glutamatergic transmission. Interestingly, when it comes to non-abdominal adult muscles, one general theme seems to be an active promoter (GAL4 driver) for the kainate-type glutamate receptor called Clumsy. The authors propose that Clumsy could be key to understanding how functional GluR complexes are assembled in adult insects.

Strengths:

(1) Documenting the types of glutamate receptors that operate in diverse insect muscle systems is important because it uncovers fundamental information.

(2) Much of the prior research focus has been on how the body wall muscle tetramers assemble and operate. It is a strength to demonstrate the other receptor solutions used by adult NMJs.

(3) The work uses GAL4 drivers and immunohistochemistry (when possible) in combination to draw conclusions.

(4) The muscle anatomical analyses are of high quality. This allows the research group to reach refined conclusions.

(5) The confocal-level images of synaptic active zones and their apposed glutamate receptor clusters are of high quality.

Weaknesses:

(1) There is a strawman argument that is used repeatedly to highlight the significance of the work. The argument implies that the field broadly assumes (or “tacitly” assumes) that the larval body wall glutamate receptor composition extrapolates to all muscles of the fly, including the adult. This reviewer cannot find evidence that this assumption or argument has been explicitly promulgated by others. More likely, others have not examined these muscles directly, and thus, they have not speculated one way or the other.

The reviewer is correct that we could not find a paper stating explicitly that "the adult NMJ is molecularly identical to the larval NMJ," and we have revised the text to avoid implying that such an explicit claim exists. However, we do argue that larval body-wall findings are routinely presented as properties of "the *Drosophila* NMJ," "the fly NMJ," or "*Drosophila* muscle". That unqualified generalization is what has led readers (ourselves included) to extrapolate the canonical larval architecture to the adult.

Many concrete examples can be found in the literature. He and Dickman (2025, Curr Opin Neurobiol) state that "two subtypes of ionotropic glutamate receptors (GluRs) mediate postsynaptic currents in *Drosophila* muscle, GluRIIA- and GluRIIB-containing," and describe their regulation by Neto as a general feature of "the fly NMJ". The word "adult" does not appear in the review. Chou et al. (2020, Neural Development) similarly assert that "at the *Drosophila* NMJ, iGluRs are heterometric tetramers composed of three invariant subunits, GluRIII/GluRIIC, GluRIID, and GluRIIE, as well as one of either GluRIIA or GluRIIB". A highly cited review of these receptors is titled "Glutamate receptors at the *Drosophila* neuromuscular junction" (DiAntonio, 2006) yet describes only the larval body-wall system, and Harris and Littleton (2015, Genetics) introduce the larval NMJ as "a well-established model glutamatergic synapse" before discussing "the fly NMJ" throughout. Notably, we could not find a review paper about *Drosophila* neuromuscular control that discussed differences in receptor composition between larval and adult muscles.

This assumption is related to the genesis of this project, which came about due to the failure of pharmacological tools used in the larva (e.g., philanthotoxin) to block synaptic transmission at adult fly muscles. We (the Tuthill Lab) contacted several principal investigators studying adult fly motor control and within the larval NMJ field (including now co-author Dion Dickman) and asked for advice about why these drugs were not effective in paralyzing leg muscles. Nobody suggested that this could be because adult muscles use different glutamate receptors than the larvae. Indeed, it was only years later when we noticed a high level of GluCl expression in the FlyCellAtlas data that we realized it could be due to differences in receptor expression.

We agree with the reviewer that adult muscle has rarely been examined directly (Rivlin et al., 2004 being the principal exception, which we cite and now discuss at greater length). Our argument is that this gap has been filled by an untested extrapolation from larvae rather than by data. Of the ~50 papers citing Rivlin et al. (2004), we could not find any that compare glutamate‑receptor subunit expression between larval and adult muscle. Follow‑up work pursued NMJ morphology, remodeling, adhesion molecules, and transporters, leaving the receptor question open.

Overall, we respectfully disagree with the reviewer that this argument is a strawman, because it reflects our lived experiences as active investigators of adult and larval fly motor control. However, we take the reviewer’s point. We have removed language implying that an explicit model was being overturned, and we now frame the assumption as implicit and not universally held. We note only that the assumption was operative in practice: this project began when pharmacological tools that reliably block transmission at the larval NMJ (e.g., philanthotoxin) failed to paralyze adult leg muscles, and when we consulted colleagues who work on the larval NMJ and adult fly motor control, differences in receptor composition were not among the explanations offered. We recognize this is an anecdote rather than evidence, and we have kept it out of the manuscript.

(2) Related - to the extent that there has been any tacit assumption about GluRIIC/D/E-anchored receptors being ubiquitous among adult muscles, tacit doubt was raised by Rivilin et al., 2004 (cited by the authors but not as a source of doubt) and by RNAseq datasets like FlyAtlas from 2022 (replicated in Figures s11 and s12). To be clear, the current analysis is better than a bulk transcript analysis from adult tissues. But rather than “overturning” a field or being paradigm-shifting, the current data seem confirmatory of FlyAtlas - and confirmatory of Rivlin et al., 2004, which explicitly concluded that larval and adult NMJs were different

Rivlin et al. did not examine GluRIIC, D and E, or most of the other glutamate receptor subunits. Nevertheless, we revised the beginning of our Discussion (pg 8) to position our work in the context of their important findings:

In 2004, Rivlin and colleagues conducted a careful survey of NMJs in seven muscles in the adult *Drosophila* thorax. They established that adult NMJs are morphologically distinct from larval NMJs, and that GluRIIA and GluRIIB are expressed in muscle-specific subsets, with some muscles mysteriously lacking both. Importantly, they speculated that some adult muscles must be using different glutamate receptor subunits (which were unknown at the time) to build synapses. This problem has remained unexplored for the last two decades.

As the reviewer requested, we have removed "overturning"/"paradigm-shifting" language throughout. But we respectfully disagree that our analysis is “confirmatory” of the Fly Cell Atlas. The Fly Cell Atlas (Li et al., 2022) does not mention glutamate receptors, or the NMJ, anywhere in its analysis. It is a general single-nucleus transcriptomic resource whose adult-muscle glutamate-receptor content was never extracted, curated, or interpreted. Extracting meaning from the dataset required posing the question, curating the relevant cells and genes, and analyzing them, which is itself a meaningful scientific contribution.

(3) One can draw expression-level conclusions from these data. But genetic tests (e.g., would clumsy losses of function impair leg muscles?) could help the authors and the field draw stronger conclusions about the roles of some of these glutamate receptor gene products. The current dataset falls short of definitively establishing the function of alternate glutamate receptor modules.

We intend to perform functional and behavioral experiments with clumsy loss-of-function flies. However, this work will require creation of new genetic reagents and behavioral experiments that make it beyond the scope of this manuscript. We intend to address this important topic in a future paper.

(4) The confocal synaptic images are of high quality. They are good enough that one could analyze how well Brp directly apposes a specific glutamate receptor subunit for all the associated imaging data underlying Figures S1-S8. No such analysis is done, but understanding what components seem to directly oppose the site of release could lead to better conclusions.

In all tissues where IIA-E are expressed at the NMJ, the protein localization is directly adjacent to Brp (see representative images in Figure 2B and s5-8). This localization is often clear in single z-slices. We attempted to perform more detailed analyses, like measure the distance between Brp and GluR puncta, but found it very challenging due to the 3D orientation of the NMJ structure. We added a sentence to page 4 of the manuscript describing what we see and the overall similarity of IIA-E localization.

Overall Assessment and Discussion:

The data in this study are of high quality, and the results support the main conclusion: adult muscle glutamate receptor clusters do not recapitulate the “canonical” larval body wall clusters. This is important, and the data stand on their own. That is the most important part. This reviewer does have suggestions on how to put the current work in proper context; the current draft appears to overstate the novelty of the findings. Additionally, some sentences need editing for accuracy. None of those concerns impeach the excellent foundational data.

**Reviewer #2 (Public review):**

Summary:

This manuscript presents a broad survey of glutamate receptor composition at the neuromuscular junction in *Drosophila* across developmental stages and muscle types. The topic is clearly important, and the central observation-that adult muscles differ substantially from the canonical larval NMJ-is interesting and potentially impactful. The dataset is extensive and will likely be of value to the community. However, in my view, there are significant limitations in how the data are generated and interpreted, which at present reduce the strength of the conclusions.

Strengths:

The study addresses a relevant and timely question and provides a large and systematic dataset. The finding that adult muscles diverge from larval NMJ organization is compelling and challenges a widely held assumption in the field. The breadth of approaches, including genetic reporters, immunohistochemistry, endogenous tagging, and transcriptomic data, is, in principle, a strong aspect of the work and allows for a broad overview of receptor expression across tissues and developmental stages. Even in its current form, the manuscript provides useful descriptive information that will be of interest to the community.

Weaknesses:

A major concern is the reliance on a heterogeneous combination of detection methods (GAL4 reporters, antibody staining, endogenous tagging, and RNA), which are treated largely as equivalent lines of evidence. These approaches differ substantially in what they measure and in their sensitivity and specificity. While convergence across methods can in principle be convincing, here this convergence is often inferred from the shared absence of signal. This is problematic because all methods used are susceptible to false negatives for different reasons. As a result, the repeated conclusion that specific GluR subunits are “absent” from adult muscles, including those previously considered essential, is not fully justified by the data presented.

This issue is not only theoretical. The manuscript itself seemingly contains examples where methods disagree, demonstrating that detection is incomplete and method-dependent. These discrepancies could be better integrated into the interpretation. Instead, negative results across methods are often taken as strong evidence for absence, which overstates the certainty of the findings.

In addition, antibody validation appears to rely largely on prior work in larval tissue. Given the structural and biochemical differences in adult muscles, it is not clear that staining performance is equivalent, particularly in cases where the signal is weak or undetected. This further complicates the interpretation of negative results.

More generally, the manuscript moves in several places from descriptive observations to functional or mechanistic implications that are not directly supported. The suggestion that adult muscles operate with fundamentally different receptor assemblies is intriguing, but remains speculative without functional validation. At a minimum, the distinction between observation and interpretation should be made more explicit.

I thus think that the current conclusions need to be more carefully constrained. Ideally, the study would be strengthened by at least one functional experiment, such as electrophysiological recordings from adult NMJs or perturbation of candidate receptors like GluClα or Clumsy. This would help to anchor the expression data in synaptic function.

We thank the reviewer for a careful and constructive critique. We agree that inference from the absence of signal requires caution, and we have revised both the wording and the framing of the manuscript substantially in response. We would also like to draw attention to a feature of our dataset that we did not previously make explicit, and which we believe speaks directly to the core concern: a number of our negative results are internally controlled by positive signal from the same reagent, in the same tissue, in the same preparation.

(i) In the adult femur, anti-GluRIIB labels NMJs on tibia extensor fibers but not on accessory tibia flexor fibers within the same bisected femur, processed, stained, and imaged together (Figure 3C-D).

(ii) Anti-GluRIIC shows the reciprocal pattern in the same preparations (Figure 3E-F). The failure to detect GluRIIC in the tibia extensor therefore cannot be attributed to a general failure of that antibody in adult tissue, to fixation, or to reagent penetration, because the same antibody labels NMJs tens of microns away in the same section.

(iii) In indirect flight muscle, where we detected none of the five GluRII subunits, we reliably detected Brp, Neto-β::sfGFP, and GluClα:V5 at and around NMJs in the same preparations (Figures 2B, 2D, 4C). Reagent access, epitope preservation, and NMJ identification are therefore all demonstrated in the same tissue where our negative results are strongest.

These controls do not exclude the possibility that receptors are present below our detection threshold, and we have accordingly replaced "absent" with "not detected" throughout the manuscript. They do, however, argue that the muscle-specific differences we report reflect genuine biological variation rather than method failure. We have added a paragraph to the Discussion making this argument explicitly, alongside a new paragraph describing the discrepancies between methods, and we have added a description of antibody validation to the Methods.

In summary, this is an interesting and potentially important study, but the current manuscript somewhat overinterprets heterogeneous and partly indirect evidence. It will already be useful in its present form, but could be more convincing if the authors more rigorously account for methodological limitations and moderate their claims accordingly.

**Reviewer #3 (Public review):**

The Sustar et al. manuscript catalogs glutamate receptor composition across distinct *Drosophila* NMJs: larval and adult abdominal NMJs, as well as NMJs on adult leg and flight muscles. This work is important and probably overdue. The larval NMJ is the exemplar NMJ in this system, and the identity of “essential” and “alternative” subunits at this stage is assumed by many to hold across developmental stages and NMJ types. Here, the authors show that there is surprising diversification among NMJ types and that the notion of essential/alternative subunits only holds true at larval NMJs.

The study will generate interest in the Clumsy GluR subunit, which has not been well-characterized at all, but is widely expressed at adult NMJs. They also find striking extrasynaptic expression of glutamate-gated chloride channel GluRClalpha in adult leg and flight muscles, raising questions about its role. The study is interesting, logical, and well-written. The figures are clear, and the discussion was particularly thoughtful. I have a couple of comments that the authors could consider.

(1) They cite Rivlin et al., (2004) in the Introduction as the sole previous study to investigate the molecular composition of adult NMJs, but do not mention this work again. In the Discussion, it would be helpful to compare/contrast their finding with those of the earlier work.

Thank you for the suggestion. We have revised the beginning of our Discussion (pg. 8) to position our work in the context of Rivlin et al.’s important findings.

(2) Were these analyses done in adults of consistent ages? It seems possible that the GluR subunit composition could be different in very young adults or in aged flies. The age of the animals should be mentioned in the Methods.

Thank you for noticing this gap. We added age of flies to the Methods section, including the important detail that in cases where we failed to detect any GAL4 signal (e.g., GluRIID-GAL4), we additionally imaged very young and very old flies (0-2 hours old and 20-day-old). It did not change the results.

(3) The broad expression of GluCl:V5 in adult leg and flight muscles is surprisingly robust and appears to light up the edges of all muscle fibers. Would the authors comment on the controls that were done to ensure that this staining is real and specific to animals carrying that V5 endogenous tag?

We added a sentence to the results section (pg 8) to highlight the V5 negative staining controls that are shown in Figure s15.

(4) The snRNAseq data in Figure S12 differ a bit from the IHC/GAL4 data summarized in the table in Figure 2. In particular, the data suggests that Ukar and Grik are widely expressed in adult muscles. Is there a reason not to include an “snRNA seq” column in Figure 2 alongside the data from GAL4 lines and IHC? To my mind, it is about as reliable as GAL4 lines that often capture only a subset of the full expression pattern. In this case, the snRNAseq data suggest that Ukar/Grik are likely at adult flight muscle NMJs, which might be important since NMJ was negative for everything except Neto-beta by IHC.

We liked your suggestion to put these data together in one figure, and we tried to incorporate this during our revisions. However, because of the difficulty of combining multiple types of data into one table, we ultimately decided to keep them separate to avoid any confusion. The GAL4 analysis is qualitative (no expression, weak expression, strong expression) in percent of muscles. The RNA-seq data is quantitative and measured in fraction of muscle cells. Figure s13 now shows RNA-seq results in a table format that is parallel to the table with GAL4 results in Figure 2.

We also added a paragraph to the end of the discussion to mull several discrepancies in results (e.g. Grik and Ukar) when using different methods.

We did observe some discrepancies across the different experimental approaches. For GAL4 reporter lines, we used T2A gene trap lines where possible (for 15 of the 16 GluR genes) to determine receptor expression. However, some of these reporters were extremely weak and may have resulted in false negatives. For example, we did not detect Grik-GAL4 or Ukar-GAL4 expression in any tissue (Figure 2C), despite RNA-seq evidence that these are expressed robustly in adult muscles (Figures s11, s12). We do not have antibodies or protein reporters for these genes to help resolve this. Another discrepancy was with GluRIIE. In this case, antibody staining and RNA-seq did not detect GluRIIE in flight muscles (Figures s8, s11), while the GAL4>GFP reporter showed extremely faint signal in the flight muscles. Because of the marginal signal and the discrepancy with the other methods, we believe this could likely be a false positive. The table in Figure 2 does not effectively capture the nuance of this otherwise confusing result. Overall, however, we found that results from different approaches generally agreed and revealed intriguing differences between different muscle types.

**Recommendations for the authors:**

**Reviewer #1 (Recommendations for the authors):**

Most of these recommendations are for text edits. Where experiments are noted (#8 and #9 below), they could be helpful, but they are not essential because the study has value as is.

(1) It would be appropriate to scrub the entire manuscript of statements that say that the work is overturning an incorrect model about larval GluRII receptor tetramers working the same way in adult muscles. First, it is not necessary. But more importantly, by this reviewer’s reading, there is not an explicit model promoted by others that the larval body wall findings extrapolate to other muscles. The better way to frame the work is to state that it clearly shows the field has a lot of work to do to properly characterize glutamate receptor composition and function in *Drosophila* adults. Places in the text that the authors should edit include:

- “...challenging assumptions about the uniformity of neuromuscular function” (Abstract)

We deleted this clause.

- “...it is often tacitly assumed...This extrapolation overlooks...during metamorphosis.” (Introduction)

New wording: … “…it has commonly been assumed…” (Abstract) and “…we and others assumed…” (Intro)

- “(With one notable exception, Rivlin et al., 2004)” (Introduction). This reference deserves more than an obscure parenthetical because it explicitly concluded that larval and adult NMJs differ.

As described above, we revised the beginning of our Discussion (pg 8) to position our work in the context of Rivlin et al.’s important findings.

In 2004, Rivlin and colleagues conducted a careful survey of NMJs in seven muscles in the adult *Drosophila* thorax. They established that adult NMJs are morphologically distinct from larval NMJs, and that GluRIIA and GluRIIB are expressed in muscle-specific subsets, with some muscles mysteriously lacking both. Importantly, they speculated that some adult muscles must be using different glutamate receptor subunits (which were unknown at the time) to build synapses. This problem has remained unexplored for the last two decades.

- “Collectively, these results overturn the assumption of NMJ uniformity...” (Introduction)

New wording: Collectively, these results reveal a diversity in NMJ composition”

- “Our findings challenge the assumption...” (Conclusions)

We deleted this clause.

(2) (Related) The authors quibble in a couple of places that the GluRIIC/D/E subunits have been “previously considered essential for viability and NMJ function.” This reviewer’s understanding of those subunits has always been that they are essential for life, and indeed, they are essential because null mutants arrest as embryos. The places where the text seems to combine viability and NMJ function (including the Abstract) as incorrectly assigned roles for these subunits, should be more carefully edited. This reviewer agrees with the “NMJ function” conclusion since the authors are considering so many NMJs. Viability still holds.

We agree with the reviewer, and we thank them for catching an imprecision that recurred throughout the manuscript. GluRIIC, GluRIID, and GluRIIE are essential for viability, and nothing in our data contradicts that conclusion.

We think the point we were trying to make is narrower and sharper when stated precisely. Null mutants for these subunits arrest as embryos — that is, they die before the adult leg and flight muscles are formed. Those experiments therefore established a requirement for viability and for larval NMJ function, but they could not, in principle, have tested whether the same subunits are required at NMJs that arise only during pupal development. Our data do not revise the viability conclusion. They show that the subunit requirement established at the larval body-wall NMJ does not generalize to every NMJ in the animal.

We have decoupled "viability" from "NMJ function" throughout the manuscript (Abstract, Introduction, Discussion) and have added a sentence to the Discussion making the developmental logic explicit:

Importantly, because these mutants die before metamorphosis, these experiments established a requirement for viability and for larval NMJ function, but could not test whether the same subunits are required at adult NMJs, which are formed during pupal development.

(3) “...the physiology and molecular architecture of adult fly NMJs remain largely unexplored...” The authors cite the Rivilin paper that examined the prothorax. But physiology has certainly been examined at DLMs as well as proboscis NMJs.

As the manuscript says on page 2, the topic is largely unexplored compared to the larva. The Rivlin paper is an excellent contribution but was conducted over 20 years ago with more limited genetic reagents. There were also no follow-up studies. In the same amount of time, over 1000 papers have been published regarding the *Drosophila* larval NMJ.

The reviewer is correct that physiology has been performed in adult DLMs and proboscis muscles. These studies, however, have focused on other biological questions and did not investigate the makeup of the NMJ or manipulate glutamate receptors with pharmacology or genetic tools. Our study reveals the need for such experiments.

(4) “Surprisingly, we found that none of the primary GluRII subunits was strongly expressed in adult indirect flight muscles...” This sentence was difficult to parse when simultaneously examining the large dot for GluRIIE-Gal4 in the IFMs (Figure 2C), until the realization that the blank IHC result was driving the conclusion (no expression). The authors may want to delineate Gal4 vs. IHC for each dataset to avoid confusion for readers.

We agree with the reviewer that this result is confusing. GluRIIE-GAL4 expression was a case in which a dot in the table didn’t capture the subtlety of the result. The GluRIIE-GAL4 was expressed very faintly in the IFM, to a degree that we almost called it negative.

We added a paragraph to the Discussion in which we reflect on some data discrepancies:

We did observe some discrepancies across the different experimental approaches. For GAL4 reporter lines, we used T2A gene trap lines where possible (for 15 of the 16 GluR genes) to determine receptor expression. However, some of these reporters were extremely weak and may have resulted in false negatives. For example, we did not detect Grik-GAL4 or Ukar-GAL4 expression in any tissue (Figure 2C), despite RNA-seq evidence that these are expressed robustly in adult muscles (Figures s11, s12). We do not have antibodies or protein reporters for these genes to help resolve this. Another discrepancy was with GluRIIE. In this case, antibody staining and RNA-seq did not detect GluRIIE in flight muscles (Figures s8, s11), while the GAL4>GFP reporter showed extremely faint signal in the flight muscles. Because of the marginal signal and the discrepancy with the other methods, we believe this could likely be a false positive. The table in Figure 2 does not effectively capture the nuance of this otherwise confusing result. Overall, however, we found that results from different approaches generally agreed and revealed intriguing differences between different muscle types.

(5) “GluClalpha in adult muscles establishes the molecular identity of inhibitory glutamate responses described decades ago in other insects...” This reviewer really likes this idea. But the current data fall far short of the conclusion in this sentence.

You are correct. We tempered this statement to “The identification of extrasynaptic GluClα in adult muscles establishes a possible mechanism for inhibitory glutamate responses described decades ago […]”

(6) “One past study (Han et al., 2015) explored many combinations....” Nothing in this sentence is untrue. This reviewer just wanted to point out that one interesting observation from the Han paper was that an A/E combination alone yielded significant function in the heterologous system. To the extent that A and B have been described as competitive for the same C/D/E core, it is not a stretch to imagine that a B/E combination would have significant function, just like A/E. Or B/D/E as well.

We thank the reviewer for this observation, which we think materially strengthens the plausibility of our interpretation, and we have incorporated it into the Discussion. Han et al. (2015) found that GluRIIA and GluRIIE together were sufficient to reconstitute functional receptors in a heterologous system, without GluRIIC or GluRIID. Because GluRIIA and GluRIIB are thought to compete for the same position within the tetramer, there is no obvious reason a GluRIIB/IIE or GluRIIB/IID/IIE complex should not also be functional. This provides a concrete precedent for exactly the kind of non-canonical combinations our expression data imply — for example, GluRIIB in the absence of detectable GluRIIC in tibia extensor fibers. We have added the following to the Discussion (p. 9):

Notably, Han et al. (2015) found that GluRIIA and GluRIIE alone were sufficient to reconstitute functional receptors, without GluRIIC or GluRIID. Because GluRIIA and GluRIIB are thought to occupy the same position within the tetramer, this raises the possibility that GluRIIB-containing complexes lacking GluRIIC — such as the GluRIIB/IID/IIE combination implied by our data in the tibia extensor — could likewise be functional. Neto-β, which is required for receptor gating and synaptic clustering (Han et al., 2024; Kim et al., 2012), is expressed at all adult NMJs we examined (Figures 2D, 3B), so the auxiliary subunit requirement for such complexes would be satisfied. Direct tests of these subunit combinations by heterologous reconstitution are an important next step.

(7) GluClalpha roles: The idea of a negative feedback controller is a good one. An alternative idea can be gleaned from the NMJ anatomy of circuits like the Mauthner Cell Escape Circuit in zebrafish. In that circuit, the fish have excitatory connections on some muscles and simultaneous control of inhibitory connections wired for other muscles to enable fast changes of direction.

We thank the reviewer for this interesting comparison. We think the Mauthner arrangement is unlikely to apply here, for two reasons. First, GluClα appears to be expressed broadly across the leg and flight muscles we examined, rather than in a subset complementary to the excitatory receptors. Second, and more importantly, GluClα is extrasynaptic: it is distributed along the periphery of the muscle fiber and is not apposed to Brp-positive active zones (Figure 4C-D). Target-specific inhibitory transmission of the kind used in the Mauthner circuit requires inhibitory receptors positioned at synapses, whereas the localization we observe is better suited to tonic or slowly varying modulation of muscle excitability. We have added a sentence to the Discussion making this distinction, since the reviewer's question is one that readers will also have. And add to the manuscript, at the end of the GluClα hypothesis paragraph in the Discussion:

We note that this arrangement differs from circuits in which excitatory and inhibitory transmission are targeted to different muscles to produce rapid directional control, as in the Mauthner cell escape circuit of larval zebrafish. While most arthropods possess GABAergic motor neurons that directly inhibit leg muscles, holometabolous insects such as *Drosophila* do not (Witten and Truman, 1998). And because GluClα is extrasynaptic rather than apposed to active zones, it is unlikely to support fast, target-specific inhibitory transmission.

Two optional experimental suggestions:

(8) The authors’ findings with Clumsy expression along with Clumsy message expression in the FlyAtlas RNAseq datasets - and finally, the unpublished work on Clumsy - all collectively suggest that it is a great target for adult knockdown. It is not required, but the authors’ conclusions would be bolstered if they were to test RNAi against Clumsy in chosen adult NMJs and check behavior (e.g., walking?) for defects.

We intend to perform functional and behavioral experiments with clumsy loss-of-function flies. However, this work will require creation of new genetic reagents and behavioral experiments that make it beyond the scope of this manuscript. We intend to address this in a future paper.

(9) The confocal synaptic images are of high quality. They are good enough that one could analyze how well Brp directly apposes a specific glutamate receptor subunit for all the associated imaging data underlying Figures S1-S8. No such analysis is done, but understanding what components seem to directly oppose the site of release could lead to better conclusions.

In all tissues where IIA-E are expressed at the NMJ, the protein localization is directly adjacent to Brp (see representative images in Figure 2B and s5-8). This localization is often clear in single z-slices. We attempted to perform more detailed analyses, like measure the distance between Brp and GluR puncta, but found it very challenging due to the 3D orientation of the NMJ structure. We added a sentence to page 4 of the manuscript describing what we see and the overall similarity of IIA-E localization.

**Reviewer #2 (Recommendations for the authors):**

The authors should revise the wording throughout to more clearly distinguish between “absence” and “not detected,” in particular for statements regarding GluRII subunits in adult muscles. In the current form, some claims appear stronger than supported by the methods.

Thank you for this important suggestion. We replaced “absence” with “not detected” throughout the manuscript. We also note this distinction explicitly in the Discussion.

It would also improve clarity if the different experimental approaches (GAL4, antibody staining, tagging, RNA-seq) were presented more explicitly separated, rather than combined into a single readout. This would allow a better assessment of the evidence.

We have kept the Results organized by finding rather than by technique, because the majority of our results were concordant across approaches and a technique-by-technique presentation would fragment each biological conclusion across four sections. However, we agree that the evidence should be separable by the reader, and it now is: Figure 2C reports GAL4 and IHC in separate columns, Figure s13 presents the RNA-seq results in a parallel table format, and the new Discussion paragraph identifies each case in which the methods disagreed and states which result we consider likely to be a false positive or false negative. Where a single method underlies a conclusion, we now say so in the text.

The discrepancies between methods (e.g., RNA-seq vs GAL4 expression) should be discussed more directly, and the possibility of false negatives, especially for antibody staining in adult tissue, should be acknowledged more clearly.

Thank you. Other reviewers also noticed this weakness of our manuscript, and as noted above, we added a paragraph in the discussion to highlight several important discrepancies in methods and possibilities of false positives.

A functional experiment is not strictly required, but would clearly strengthen the manuscript. For example, electrophysiology at adult NMJs or perturbation of candidate receptors (e.g., GluClα or Clumsy) would help to link expression to function.

We intend to perform functional and behavioral experiments with clumsy loss-of-function flies. However, this work will require creation of new genetic reagents and behavioral experiments that make it beyond the scope of this manuscript. These experiments and the accompanying behavioral analysis will form a separate study, which will be the foundation of a future paper.
